# Dosimetric characteristics of a new linear accelerator under gated operation

**DOI:** 10.1120/jacmp.v7i1.2162

**Published:** 2006-02-21

**Authors:** Sergey Kriminski, Alex N. Li, Timothy D. Solberg

**Affiliations:** ^1^ Department of Radiation Oncology David Geffen School of Medicine at UCLA Los Angeles California 90095; ^2^ Department of Medical Physics Memorial Sloan‐Kettering Cancer Center New York New York 10021; ^3^ Banner Desert Medical Center Mesa Arizona 85202; ^4^ Jonsson Comprehensive Cancer Center Los Angeles California 90095; ^5^ University of Nebraska Medical Center Omaha Nebraska 68102 U.S.A.

**Keywords:** respiratory gating, Siemens ONCOR Avant-Garde, Kodak EDR2 and X-Omat V films, dosimetry

## Abstract

Respiratory gated radiotherapy may allow reduction of the treatment margins, thus sparing healthy tissue and/or allowing dose escalation to the tumor. However, current commissioning and quality assurance of linear accelerators do not include evaluation of gated delivery. The purpose of this study is to test gated photon delivery of a Siemens ONCOR Avant‐Garde linear accelerator. Dosimetric characteristics for gated and nongated delivery of 6‐MV and 15‐MV photons were compared for the range of doses, dose rates, and for several gating regimes. Dose profiles were also compared using Kodak EDR2 and X‐Omat V films for 6‐MV and 15‐MV photons for several dose rates and gating regimes. Results showed that deviation is less than or equal to 0.6% for all dose levels evaluated with the exception of the lowest dose delivered at 25 MU at an unrealistically high gating frequency of 0.5 Hz. At 400 MU, dose profile deviations along the central axes in in‐plane and cross‐plane directions within 80% of the field size are below 0.7%. No unequivocally detectable dose profile deviation was observed for 50 MU. Based on the comparison with widely accepted standards for conventional delivery, our results indicate that this LINAC is well suited for gated delivery of nondynamic fields.

PACS numbers: 87.56‐By, 87.66‐Cd, 87.66‐Jj

## I. INTRODUCTION

The objective of radiation therapy is to maximize the dose to the target volume while sparing the normal tissue as much as possible. With the current geometrical precision achieved by multileaf collimators, respiration‐induced organ motion may be a major source of error in the delivered dose distribution.^(^ ^1^ ^–^ ^6^ ^)^ Motion, which may have an amplitude^(^ ^3^ ^,^ ^7^ ^–^ ^19^ ^)^ up to 2 cm to 3 cm at normal breathing, leads to blur and deformation of the dose distribution.^(^ ^2^ ^,^ ^20^ ^)^

Gated delivery of radiation is a potential technique for reducing the effects of respiratory motion.^(^ ^1^ ^,^ ^2^ ^,^ ^5^ ^,^ ^10^ ^,^ ^11^ ^,^ ^13^ ^–^ ^15^ ^,^ ^19^ ^,^ ^21^ ^,^ ^22^ ^)^ Internal organ displacement appears to correlate with external surrogates of the respiratory motion. Such a correlation has been observed by several researchers: during free respiration,^(^ ^7^ ^,^ ^10^ ^,^ ^13^ ^)^ during coached respiration, when patients receive audio and/or visual feedback,^(11)^ and during respiration with breath‐hold.^(23)^ Therefore, in most cases some type of an external surrogate can be selected to properly gate radiation delivery for these techniques. It should be mentioned that a phase shift was observed for some patients between surrogate and diaphragm motions,^(19)^ and, therefore, radiographic surveillance may be recommended. Presumably, such surveillance should be carried out throughout the entire course of treatment because of possible interfraction variation.^(11)^

Although gating may often be the best approach to deal with respiratory motion, current commissioning and quality assurance of linear accelerators ordinarily do not include evaluation of gated radiation delivery. An extensive evaluation of the performance during gating delivery is necessary prior to such use. Such evaluations were previously performed on two types of LINACs: Novalis (BrainLAB AG, Heimstetten, Germany) and Varian^(^ ^13^ ^,^ ^24^ ^–^ ^26^ ^)^ (models Clinac 2100C/D, Varian Oncology Systems, Palo Alto, CA). Investigations on the Novalis LINAC,^(27)^ those that did not involve dynamic elements such as use of virtual wedge or dynamic multileaf collimator, showed less than 1.7% absolute dose deviation, with the exception of cases of unreasonably low dose rate and/or total dose and unreasonable gating frequencies. Static tests with Varian equipment showed slightly better results, with 0.8% deviation in absolute dose and approximately 1% flatness and symmetry deviation from the nongated delivery. For both LINACs, deviations in nondynamic delivery settings were acceptable for clinical use.

In this work, evaluation of gated photon delivery from a commercial linear accelerator (Siemens ONCOR Avant‐Garde, Siemens Medical Solutions, Concord, CA) is carried out. To our knowledge, no study of gated radiation delivery of a Siemens LINAC has been published.

Dosimetric integrity under gated and nongated delivery of 6‐MV and 15‐MV photons was compared for the following ranges: monitor units: 25 MU to 400 MU; dose rates: 75 MU/min to 500 MU/min; and for several gating regimes: 0.06 Hz to 0.5 Hz. Dose profiles were also compared using Kodak EDR2 and XV films for 6‐MV and 15‐MV photons for several dose rates, gating regimes, and field sizes. No dynamic radiation delivery was tested in our study.

## II. MATERIALS AND METHODS

The Siemens ONCOR Avant‐Garde linear accelerator used in our studies (Siemens Medical Solutions, Concord, CA, USA) was installed late 2003 and data collection was carried out late 2004 and early 2005.

### A. Mechanism of gating

In the Siemens ONCOR Avant‐Garde accelerator, the electron beam used to generate X‐rays is produced by a standing wave accelerator. In order to avoid changes in the temperature of the components of the LINAC, neither electron injection nor microwave generation is interrupted during the beam‐off state in gated operation. Rather, the beam is gated by injecting electrons asynchronously with the microwave generation. This mechanism is often referred to as “gun delay.”^(^ ^13^ ^,^ ^25^ ^)^ When this mechanism is used, energy dissipation and, consequently, the temperature in the LINAC components during gated operation are essentially unchanged from those during regular, nongated operation. Therefore, the dosimetric characteristics of a gated beam are expected to be very close to those of the beam generated without gating. In contrast to the pause state during point‐and‐shoot intensity‐modulated radiotherapy (IMRT) delivery,^(^ ^28^ ^–^ ^30^ ^)^ no adjustment in the rf power is done during the pause state in gated delivery on this LINAC.

Gating signals were generated using a PC equipped with a commercial signal generator (PCI‐20428W‐3A, Intelligent Instrumentation, Tucson, AZ). The precision in gating pulse length was approximately 2 ms. Two types of measurements were carried out. In the first, the duty cycle—the fraction of time when the beam is on—was one‐half the period of the signal, that is, the beam‐on time equaled the beam‐off time. Several signal frequencies, *f*, were tested: 0.0625 Hz, 0.125 Hz, 0.25 Hz, and 0.5 Hz. In the second, the duty cycle was varied while keeping the signal repetition frequency constant.

### B. Dosimetry

Dosimetric data was collected using a Baldwin‐Farmer 0.6 cm^3^ ion chamber and a Keithley 616 digital electrometer. Measurements were performed at 5 cm depth in water‐equivalent material at a source‐to‐surface distance (SSD) of 100 cm. Readings for gated delivery were compared to those for the nongated operation. Because of the relative character of the measurements, raw data from the ion chamber were used. Each measurement was repeated a minimum of three times in order to estimate statistical error, which was calculated following the standard convention:

(1) $$\delta C=\sqrt{\sum_{i=1}^{n}\frac{{\left(C_{i}-C\right)}^{2}}{n-1}},\;\;C=\sum_{i=1}^{n}\frac{C_{i}}{n},$$

where $C_{i}$ is the charge measured, and *n* is the number of measurements done. Note that the error corresponds to the standard deviation of a single measurement. Since we are interested only in the comparison of gated to nongated delivery, we introduce the normalized difference $\varepsilon_{g}$ between the two:

(2) $$\varepsilon_{g}=(C_{g}-C_{\text{ng}})/C_{\text{ng}},\;\;\delta \varepsilon_{g}=\sqrt{\delta C_{g}^{2}+\delta C_{\text{ng}}^{2}}/C_{\text{ng}},$$

where the subscript “g” denotes gated delivery and “ng” the nongated delivery.

Measurements for different numbers of monitor units and for different dose rates (MU/min) were performed. The parameters evaluated are summarized in Tables 1 and 2.

**Table 1:** Different LINAC settings and gating regimes studied in this work. Beam‐off duration was one half the period of the signal, that is, beam‐off time equals beam‐on time in the measurements presented in this table. Both photon energies, 6 MV and 15 MV, and four different gating signal repetition frequencies were tested: 0.0625 Hz, 0.125 Hz, 0.25 Hz, and 0.5 Hz.

Dose (MU)	Dose rates (MU/min)
25	500,^a^ 300, 150, 75
50	500,^a^ 300, 150, 75
100	500,^a^ 300, 150
200	500,^a^ 300, 150^b^
300	300^b^
400	500,^a^ 300

^a^ 15 MV only

^b^ 6 MV only

**Table 2:** Different LINAC settings and gating regimes studied in this work. Twenty‐five percent (signal 1) and 75% (signal 2) beam‐off times were used in the measurements presented in this table. The gating signal repetition frequency was 0.25 Hz. Both photon energies, 6 MV and 15 MV, were tested.

Dose rate (MU/min)	500^a^	300	150	75
Dose (MU)	200	200	50	50

^a^ 15 MV only

### C. Dose profile

Film profiles were taken at 10 cm depth in water‐equivalent material at a 100 cm SSD. All data were obtained at 0° collimator and gantry positions. Throughout the paper the following notations for the scan directions are used: in‐plane scans are parallel to the axis of gantry rotation with the positive direction being away from the gantry; cross‐plane scans are rotated 90° clockwise (as viewed from the top) with respect to the in‐plane scans.

Kodak EDR2 film was used for the relative dosimetric studies. It has a much lower sensitivity to the low‐energy portion of spectrum and has better precision compared to the traditionally used Kodak X‐Omat V film.^(^ ^31^ ^,^ ^32^ ^)^ The maximum linear field size (20 cm) was limited by the size of the films used. Films were processed 5 to 30 min after each exposure. In order to minimize the local noise inhomogeneity effect, data were convolved with 2D Gaussian with σ=0.2cm after scanning.

**Figure 1:**
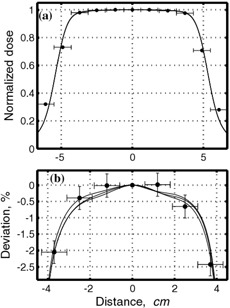
Comparison of dose profiles measured by film and by ion chamber for nongated delivery. In‐plane direction normalized to the center for 6‐MV photons for a $10\times 10\;{\text{cm}}^{2}$ field is shown. (a) Film data (solid lines) and ion chamber reading (solid circles) normalized to the value at the center. Film data are convoluted with Gaussian with σ≈5mm (approximately one‐quarter the length of the ion chamber). (b) Enlarged top segment of (a); for convenience percent deviation of normalized data from 1 is shown.

Two different fields, $10\times 10\;{\text{cm}}^{2}$ and $20\times 20\;{\text{cm}}^{2}$, were imaged for six different gating regimes (0.625 Hz, 0.125 Hz, 0.25 Hz, and 0.5 Hz for the 50% duty cycle, and the 25% and 75% duty cycles at 0.25 Hz) and for nongated delivery. Four hundred monitor units were used for film exposures at 300 MU/min and 500 MU/min for 6‐MV and 15‐MV, respectively. In order to determine errors associated with positional and dose irreproducibility, three films were obtained for nongated delivery of a $10\times 10\;{\text{cm}}^{2}$ field at 6 MV. In addition, for comparison, ion chamber profiles were also collected at 10 cm depth in water‐equivalent material. Errors were calculated as described in Eq. (2), except that data from the center were used as a reference instead of data from nongated delivery. Horizontal error bars are determined by the geometric size of the ion chamber and are half the radius (r=0.31cm) in the cross‐plane direction and one‐quarter the length (l=2.4cm) in the in‐plane direction. As seen in Fig. 1, ion chamber readings and film data agree well with each other. The relative error (precision) in EDR2 measurements, as seen in Fig. 1, is approximately 0.3%.

The speed of EDR2 film does not allow us to perform measurements at a low number of monitor units. For that reason, several control measurements were performed using Kodak X‐Omat V film, allowing measurements at much lower doses. In order to determine errors associated with the dose irreproducibility, three films were obtained for nongated delivery, similar to the EDR2 measurements. The precision of XV film was determined to be approximately 1%, somewhat worse than that of the EDR2 film. Three gating regimes, for which we expect maximum errors, were evaluated: 0.5 Hz at 50% duty cycle, and 0.25 Hz at 25%, 50%, and 75% duty cycles. Fifty monitor units and $10\times 10\;{\text{cm}}^{2}$ fields were used for film exposures at 300 MU/min and 500 MU/min for 6 MV and 15 MV, respectively.

## III. RESULTS

### A. Dosimetry

Dosimetric comparison of gated and nongated LINAC operation for 6‐ and 15‐MV photons for 25 MU to 400 MU was performed for frequencies of 0.06 Hz to 0.25 Hz for several duty cycles.

**Figure 2:**
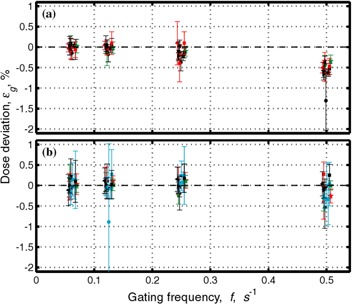
Dose deviation from dose at nongated regime normalized by the nongated dose, $\varepsilon_{g}$, for different numbers of monitor units and different dose rates as a function of gating frequency *f*. In the gating regime used, the beam was off half the time. Symbols are assigned as follows: circle: 25 MU; star: 50 MU; square: 100 MU; left‐pointing triangle: 200 MU; bottom‐pointing triangle: 300 MU; right‐pointing triangle: 400 MU. Colors were assigned as follows: cyan (15 MV only): 500 MU/min; black: 300 MU/min; red: 150 MU/min; green: 75 MU/min. Figure 2(a) shows data for 6‐MV photons and (b) for 15‐MV photons. Data are taken from the measurements shown in Table 1.

Figure 2(a) shows the deviation in dose during gated delivery, $\varepsilon_{g}$, from dose in the nongated regime normalized by the nongated dose for the 6‐MV photon beam for the 50% duty cycle. It can be observed that all doses are within 1%, with the exception of 25 MU delivered at a dose rate 300 MU/min and gated at a frequency of 0.5 Hz. The gating frequency of 0.5 Hz is unrealistically high for human respiration, but even in this case, the worst case observed for 6‐MV photons, the average dose deviation is small, below 1.5%. It should be emphasized that 1.5% deviations are observed for extreme gating regimes selected to test the limit of the accelerator. Typical human respiration frequencies are in the range of 0.13 Hz to 0.25 Hz. In this range of frequencies, the agreement between gated and nongated delivery for the 6‐MV photon beam is better than 0.5%. For delivery of 50 MU or more, the agreement in this frequency range is better than 0.25%.

Figure 2(b) shows the deviation in dose during gated delivery, $\varepsilon_{g}$, from dose in the nongated regime normalized by the nongated dose for the 15‐MV photon beam for the 50% duty cycle. Similar to the low‐energy case, all average doses are within 1%. Here, we draw the reader's attention to a large error bar (indicating standard deviation of a single measurement) of the point corresponding to 25 MU at 500 MU/min. While on average the dose is close to the nominal value in some cases the dose deviation may be as large as 2.5% to 3%. If several fractions are delivered, this error will be averaged out; at the same time, single fraction delivery of 25 MU is generally too small for clinical radiation treatment. For delivery of 50 MU or more and for the range of frequencies typical of human respiration, approximately 0.13 Hz to 0.25 Hz, agreement between gated and nongated delivery for the 15‐MV photon beam is better than 0.25%.

**Figure 3:**
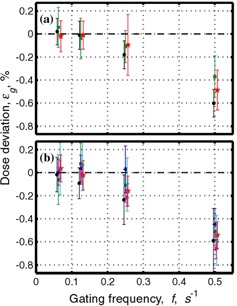
Same data as Fig. 2(a). (a) Data are averaged over all monitor units for 300 MU/min; black circles: 150 MU/min; red stars and 75 MU/min: green squares. (b) Data are averaged over all dose rates for 25 MU: black circles; 50 MU: red stars; 100 MU: green squares; 200 MU: blue left‐pointing triangle; 300 MU: cyan bottom‐pointing triangle; 400 MU: magenta right‐pointing triangle. Data are taken from the measurements shown in Table 1 for 6‐MV mode.

**Figure 4:**
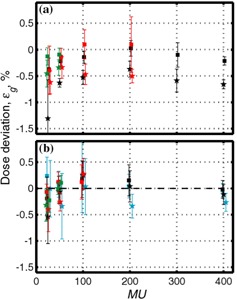
Dose deviation from dose at nongated regime normalized by the nongated dose, $\varepsilon_{g}$, for different gating frequencies *f*, and different dose rates as a function of the number of monitor units for (a) 6 MV and (b) 15 MV. Symbols were assigned as follows: star: 0.5 Hz; square: 0.25 Hz. Colors were assigned as follows: cyan (15 MV only): 500 MU/min; black: 300 MU/min; red: 150 MU/min; green: 75 MU/min. Data are taken from the measurements shown in Table 1.

Figures 3 and 4 contain identical data to Fig. 2, but are plotted differently. Figure 3 shows data averaged over different doses at the same dose rate (Fig. 3(a)) and over the same dose at different dose rates (Fig. 3(b)). As gating frequency increases, a clear trend toward poorer agreement between gated and nongated data can be observed. No such trend is observed for the 15‐MV mode (Fig. 2(b)).

Figure 4 shows the relative dose deviation from the dose delivered in the nongated regime as a function of number of monitor units. It is clearly seen that for a constant gating frequency, the relative dose deviation is larger for smaller numbers of monitor units delivered, and relatively constant above approximately 100 MU. It can also be seen that for 6 MV the dose deviation is larger for a higher gating frequency (Fig. 4(a)).

**Figure 5:**
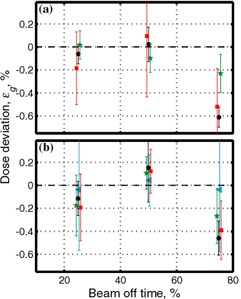
Dose deviation from dose at nongated regime normalized by the nongated dose, $\varepsilon_{g}$, versus fraction of time beam is off for 0.25 Hz gating frequency (a) for 6 MV and (b) for 15 MV. Data are taken from the measurements shown in Table 2 and, for 50% time‐off data, in Table 1. Cyan triangles correspond to 500 MU/min (15 MV only); black circles: 300 MU/min; red squares: 150 MU/min; green squares: 75 MU/min.

Figure 5 shows the dependence of dose deviation on the percentage of time when the beam is off, while keeping the repetition frequency the same. A small variation is observed increasing slightly the longer the beam is off. We speculate that for 15 MV, this effect may be due to background count in the LINAC's ion chamber, while for 6 MV the growth of the number of interruptions with increasing beam‐off time may also be important. The dose deviation observed is below 0.6% for both photon energies. Based on the results for the 50% duty cycle, we do not expect significant deviations for frequencies below 0.25 Hz.

We should emphasize that although there are some trends observed in Figs. 2 to 5, deviations are small, in most cases less than 1%, and in cases relevant to clinical situations deviations are less than or equal to 0.6%.

### B. Dose profile

**Figure 6:**
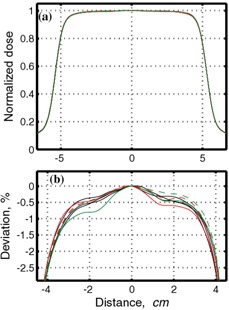
Comparison of dose profiles for gated and nongated delivery for 6‐MV photons for a $10\times 10\;{\text{cm}}^{2}$ field. Profiles are measured in the in‐plane direction and normalized to the center. (a) Relative dose. (b) Enlarged top segment of (a); for convenience normalized dose deviation from 1 was used. The black solid lines correspond to film data for nongated delivery. The red solid line denotes gated delivery at 0.125 Hz frequency with 50% duty cycle; solid green: at 0.25 Hz with 50% duty cycle. The dashed red line denotes gated delivery at 0.25 Hz with 75% duty cycle (25% of time beam was off) and dashed green at 0.25 Hz with 25% duty cycle (75% of time beam was off).

Similarly, good agreement was observed between dose profiles in gated and nongated delivery. Figure 6 compares in‐plane dose profiles taken for gated and nongated delivery for 400 MU using Kodak EDR2 film. For $10\times 10\;{\text{cm}}^{2}$ fields (for both 6 MV and 15 MV), if the dose difference between gated and nongated delivery is greater than 1% (of maximum value to which both gated and nongated profiles were normalized), the distance to agreement is not worse than 0.12 cm. Only the region of the dose profile above 20% was considered in our comparison. A similar statement can be made for a $20\times 20\;{\text{cm}}^{2}$ field: if the dose difference is greater than 1.5%, the distance to agreement is better than 0.11 cm. Along the central axes in in‐plane and cross‐plane directions within 80% of the field size the maximum deviation was below 0.4% for $10\times 10\;{\text{cm}}^{2}$ fields and below 0.7% for $20\times 20\;{\text{cm}}^{2}$ fields.

In order to study the changes of dose profiles for a low number of monitor units, Kodak X‐Omat V film was used. Fifty monitor units were delivered using the $10\times 10\;{\text{cm}}^{2}$ field (for both 6 MV and 15 MV). Since the precision of the film appeared to be comparable to the variation of the dose profiles due to gating, we were capable of only detecting the upper bounds for such variations: if the dose difference between gated and nongated delivery is more than 1.6%, the distance to agreement is not worse than 0.07 cm. Only the region of the dose profile above 20% was considered in our comparison. Along the central axes in in‐plane and cross‐plane directions within 80% of the field size, the maximum deviation was below 1.4%.

## IV. DISCUSSION AND CONCLUSION

In this work, an evaluation of gated photon delivery by a Siemens ONCOR Avant‐Garde linear accelerator was performed. Dosimetric characteristics for gated and nongated delivery of 6MV and 15‐MV photons were compared for the range of doses of 25 MU to 400 MU; the dose rates of 75 MU/min to 500 MU/min; and for several gating regimes: 0.5 Hz … 0.0625 Hz, using ion chamber. Dose profiles were also compared using Kodak EDR2 and X‐Omat V films at 10 cm depth in water at 100 cm SSD for 6‐MV and 15‐MV photons for different dose rates, field sizes, and gating regimes. No investigation of dynamic delivery, such as virtual wedge, was performed.

One question that may arise regarding usage of an ion chamber for such a characterization is whether dose rate effects are negligible. In order to assess this effect, one should consider the time structure of the beam. On the Siemens ONCOR Avant‐Garde, the X‐ray beam is generated in ~3μs pulses with the repetition frequency depending upon the dose rate. The highest repetition frequency is limited by the pulse forming network charging time of about 3 ms. This is comparable to the ion transit time (~1ms) in the ion chamber; thus, the collection efficiency may be below 100%. To assess this, the usual two‐voltage technique described in TG‐51^(33)^ was used to estimate the correction for ion collection efficiency $P_{\text{ion}}$ for the ion chamber used. This was performed at the highest dose rate, where dose rate effects are the most significant, and determined to be $P_{\text{ion}}-1=3.1\pm 0.9\times 10^{-3}$ (for 6 MV). However, only two pulses out of $N_{\text{pg}}$ ($N_{\text{pg}}$ is number of pulses per single beam‐off period ‐ “per gate”) are affected by the beam interruption. The smallest value for $N_{\text{pg}}$ occurs at the lowest dose rate and the highest gating frequency and is about 40. Thus, any difference in ion chamber readings between gated and nongated delivery caused by dose rate effects is on the order of $(P_{\text{ion}}-1)\;/\;N_{\text{pg}}<10^{-4}$ and, therefore, insignificant for our measurements.

Results for dose measurements are summarized in Figs. 2, 4, and 5. It is noted that the dose deviation is less than 1% for all doses, with the exception of 25 MU delivered at the highest dose rate and at unrealistically high gating frequency of 0.5 Hz. For clinically relevant gating frequencies and delivery of 50 MU or more, deviations were less than 0.6%.

Figure 6 shows an example of dose profiles during gated and nongated radiation delivery. For 400 MU delivery, either the agreement between gated and nongated dose profiles is better than 1.5% or the distance to agreement is better than 0.12 cm. Along the central axes in in‐plane and cross‐plane directions within 80% of the field size, deviations were below 0.7%. No unequivocally detectable dose profile deviation was observed for 50 MU delivery. Upper limits for the dose deviation were determined: along the central axes in in‐plane and cross‐plane directions within 80% of the field size the maximum deviation was below 1.4%.

These results can be compared with the requirements listed in TG‐40^(34)^ for the quality assurance of linear accelerators. For example, X‐ray output constancy of 2% or better is recommended, so that a dose deviation of 1% observed under gated operation is well within acceptable limits. Similarly, a 0.7% dose profile deviation at the central axes is well within 2% flatness (defined in TG‐24^(35)^) requirements.

Kubo et al.^(13)^ obtained comparable results for a Varian 2100C LINAC: central axis dose deviation was within 0.2% (except for numbers of monitor units smaller than 20, when deviations could be as large as 0.8%), and symmetry variations were as large as 1.1%. Similarly, Ramsey et al.^(25)^ observed less than 0.2% dose variations and less than 0.6% changes in flatness and symmetry for delivery of 5 MU or more. Hugo^(27)^ observed that the Novalis LINAC has less than 2% dose deviation, with the exception of unreasonably low dose rates and/or total dose at very high gating frequencies. To our knowledge, no other similar studies exist in the literature.

Jaw/leaf motion during delivery adds additional difficulty in gated delivery if a dynamic multileaf collimator (DMLC) or virtual wedge is used. Kubo and Wang^(24)^ analyzed the performance of a Varian 2100C with an 80‐leaf MLC using both a DMLC and a virtual wedge. Maximum dose deviations of less than 2% were observed in regions away from the field edges. A similar evaluation of gated IMRT delivery on a Novalis LINAC was less impressive, showing^(^ ^1^ ^,^ ^27^ ^)^ significant discrepancies exceeding 3% between gated and nongated operation. Duan et al.^(26)^ observed that for a Varian 21EX LINAC with a Millennium 120□ Leaf MLC, the error caused by gated DMLC delivery may be 3.7% or even larger for some unfavorable cases. A 3‐mm shift of isodose lines was observed.

Comparison to current standards in dose precision and to results for other LINACs shows that the Siemens ONCOR Avant‐Garde is well suited for gated delivery of static fields of 25 MU or more. However, gating does contribute some additional error to the dose and dose distribution, which leads to tighter requirements for LINAC tolerances. Also, simultaneous use of dynamic fields, such as virtual wedge or IMRT, with gating may lead to additional errors and may require a lower dose rate, thus increasing the treatment time.

## ACKNOWLEDGMENTS

This work supported by grant #03‐028‐01‐CCE from the American Cancer Society and by Siemens. We are greatly obliged to D. Hawkins and E. Calderon for technical help and fruitful discussions.
